# Impact of Plantar Fasciitis on Foot-Specific and Generic Health-Related Quality of Life in King Khalid University Hospital, Saudi Arabia

**DOI:** 10.7759/cureus.41912

**Published:** 2023-07-15

**Authors:** Sulaiman A Alshammari, Mohammed A Alshwieer, Saad S Dammas, Abdulaziz M Alrasheed, Mohammed A Alasmari, Mansour M Alahmari

**Affiliations:** 1 Family and Community Medicine, King Saud University Medical City, Riyadh, SAU

**Keywords:** foot health-related quality of life (fhsq), quality of life (qol), foot and ankle, fasciopathy, plantar fasciitis

## Abstract

Objectives

The objective is to assess the extent of foot-specific and generic health-related quality of life (HRQoL) impairment among individuals diagnosed with plantar fasciitis (PF) at King Khalid University, Saudi Arabia, and to determine the influence of various sociodemographic and clinical factors.

Methods

In this cross-sectional study, we administered an online survey from March to June 2023 to patients with PF at King Khalid University Hospital. This questionnaire covered sociodemographic, and clinical characteristics, and included the translated Foot Health Status Questionnaire (FHSQ). Further, patient data were obtained from hospital records between 2016 and 2023 using the E-SIHI system.

Results

We recruited 209 patients for the study. Lower FHSQ scores were found in unemployed and low-income participants indicating a worse quality of life (QoL) in the Foot Pain domain, while those aged over 40 years and with low income showed greater impairment in the General Foot Health domain. No single factor influenced the Foot Function or Footwear domains. Worse General Health and Physical Activity scores were associated with low-education and low-income participants and those who did not exercise. Women exhibited a lower QoL than men in all domains except for General Health.

Conclusion

The QoL of Saudi women with PF was similar to that of women in other countries. However, the patients in the present study reported poorer footwear scores but better General Health scores. Therefore, focusing more on proper footwear-related treatments may help improve the QoL of patients with PF.

## Introduction

The plantar fascia covers the plantar surface of the feet, supporting its longitudinal arches and absorbing shock. Plantar fasciitis (PF) is a disorder caused by degeneration of the plantar fascia and nearby tissues [1,2]. PF is a generalized term for conditions involving osseous and soft-tissue pathologies that affect the heel and cause plantar heel pain [3]. In communities, the prevalence of foot pain is estimated to be above 60%. Moreover, PF is considered the foremost source of foot pain in adults visiting an outpatient facility, with a prevalence of approximately 10% [4-6]. Its prevalence varies among community groups, with the highest prevalence in active runners and obese non-athlete patients; some sources indicate that up to 22% of runners may exhibit PF. Female to male ratio regarding this foot condition was estimated to be 2.5 [2,7-9]. PF is diagnosed clinically based on history alone; it presents with pain on the medial plantar side of the heel that worsens after rest periods, when taking the first step in the morning, or with weight-bearing activities. Initial improvements may occur while walking, but pain may increase throughout the day [10]. The pain caused by PF has been linked to changes in gait, deviations in stepping, and ultimately, internal rotation of the tibia and femur and alteration of overall foot function because walking is an integral part of daily routines [10]. Compared to healthy individuals, patients with PF have a much lower foot-specific and generic quality of life (QoL) and are more prone to experiencing symptoms of depression, anxiety, and stress [4,7,11,12]. Previous studies demonstrating the relationship between PF and QoL using the Foot Health Status Questionnaire (FHSQ) are scarce. One study compared the impact of PF on QoL between male and female patients, revealing a worse level in female patients across different FHSQ domains [7]. Additionally, two previous case-control studies demonstrated that individuals with PF have a worse foot-specific QoL than the general population [11,12]. Generic and specific self-reported health-related quality-of-life (HRQoL) instruments are available. For PF, the FHSQ score was initially designed as a foot-specific HRQoL metric to evaluate the effect of surgical treatment of common foot conditions. However, it has been validated in various podiatric disorders and can be used to assess generic HRQoL [7,11,13,14]. FHSQ has been demonstrated as the best appropriate measure of HRQoL in patients with PF [15]. The FHSQ is easy to administer and comprehensive and is one of the most frequently utilized and validated assessments of foot pain and impairment [12,13]. There is minimal research on the impact of PF on foot-specific HRQoL in Saudi Arabia, the Middle East, and/or Asia as a whole. This research sought to determine the extent of the impact of PF on the QoL of patients in King Khalid University Hospital (KKUH), Saudi Arabia. This could help healthcare professionals better understand the disease and develop more effective patient treatment plans. We also determined whether there were any significant differences in QoL among patients with PF according to demographic and clinical characteristics such as age, sex, occupation, standing time, income, comorbidities, sports involvement, and footwear. This could help identify specific areas where patients with PF require additional support.

## Materials and methods

Study design and settings

In this cross-sectional study, an online questionnaire was sent to patients at KKUH, Saudi Arabia. All participants gave consent to take part in this investigation. The Institutional Review Board (IRB) of the College of Medicine, King Saud University approved this project (E-23-7649).

Sample size

The sample size was calculated using single population proportion formula [16]:

N = Z∞2 * (P) * (1-P)/d2, 

where,

N = minimum required sample size, Z ∞ = 1.96 at a 95% level of significance, P = prevalence of PF worldwide (estimated to be 10%), d2 = degree of precision (assumed to be 5%), N = ((1.96)^2^ * (0.1) * (1−0.01))/(0.05)^2^ = 152.

Patient recruitment and procedure

Data on patients with PF were obtained from the KKUH E-SIHI system. The data from January 2016 to January 2023 were extracted using an Excel sheet with conjunctive data (file number, age, sex, date of diagnosis, diagnosis description, and phone number of the patient). Patients were selected using systematic random sampling based on the Excel sheet, and a questionnaire was sent to 800 patients with an expected 25%-30% response rate for the online survey [17]. The selected patients received the Google Form link to the online questionnaire via WhatsApp or SMS. If the patient could not use the online form, they were contacted directly via a phone call. Confirmation that the patient was still having the disease was assessed in the first section of the questionnaire by defining PF pain as “painful heel syndrome with localized pain at the heel of the foot, worst in the morning or after resting for a long time.” This was followed by the question “Do you still have the pain?”; “How severe is the pain?”; and “What is the exact site of the pain?”. Subsequently, the participants' files were checked by file number to apply the inclusion and exclusion criteria. The inclusion criteria were painful heel syndrome with localized pain at the fascia attachment with the worst pain being in the morning or after resting for a long time. Patients with previous foot trauma, deformity, or dysfunction (including neurological dysfunction), age extremities (below 18 and above 65 years), and prior foot surgery were excluded based on the study criteria. Acquiring data such as body mass index (BMI) was not feasible, as it could be obsolete in the hospital records. Attempts to schedule appointments to measure the participants' BMI were met with their unwillingness to comply.

Outcome measures

This study included four sections in the distributed forms. The first section was an informed consent form with a question to fill in the patient's phone number to verify the previously obtained data. The second section consisted of questions confirming active disease status, baseline characteristics, and sociodemographic data. The third section contained questions on foot-specific QoL, while the fourth section covered generic HRQoL. The FHSQ comprised two sections, with each further divided into four domains. The first section aimed to elaborate on the foot-specific HRQoL impairment, which included the following domains: “Foot Pain,” which consists of four questions about the foot pain experienced in the past week. The second domain is “Foot Function” inquiries about your overall foot health and how your feet may obstruct your ability to engage in daily activities. The third domain is “Footwear.” The fourth domain is “General foot health,” which discusses the activities you would engage in throughout a typical day, along with any current health limitations you may have and the extent of those limitations. The second section includes the following domains as part of the generic FHSQ: “General health,” “Physical activity,” “Social activity,” and “Vigor”; these domains evaluate the impact on general QoL and social interaction. The FHSQ was translated using the translation and back-translation method [18]. First, two multilingual health professionals translated the main English FHSQ into Arabic. Careful consideration was taken to ensure that the translation was conceptual rather than verbatim. Subsequently, a separate team of bilingual specialists, who were unaware of the original English version, retroactively translated the initial Arabic version of the FHSQ into English. The research committee also compared the items in the English translation of the Arabic FHSQ draft with their equivalents in the primary English questionnaire. This resulted in two composite scores ranging from 0 to 100, for each section. Scores closer to 100 imply higher QoL regarding foot-specific and general health, whereas scores nearer to 0 suggest a lower QoL. The baseline characteristics and sociodemographic data associated with patients with chronic plantar heel pain included sex, age, educational level, marital status, occupational status, standing, monthly income, exercise, other comorbidities, type of footwear, and sole footwear material [19]. Occupational prolonged standing was obtained as a percentage since each 10% increase in time spent standing is associated with an increased risk of developing PF [20,21]. Prolonged standing is defined as more than 50% standing of a time shift, and data are categorized upon that [22]. Furthermore, the type and duration of exercise were reported in total hours per week. They were divided into those who exercised less than or at least 2.5 hours per week and those who did not exercise, following the World Health Organization's recommendation. Moreover, the type of footwear and cushioning used in the shoes were listed according to the Footwear Assessment Tool and provided with a picture showing an example of each type [23].

Statistical analysis

To obtain these FHSQ scores accurately, the data were entered into the FHSQ Data Analysis Software Version 1.04. We analyzed the data utilizing SPSS version 26.0 (IBM Corp., Armonk, NY). To describe quantitative variables, mean, standard deviation, median, and interquartile range (IQR)) were used, whereas frequencies and percentages were used for the categorical variables. For bivariate statistical analysis, the investigators conducted the Kruskal-Wallis and Mann-Whitney U tests. Cronbach's alpha was obtained for each of the FHSQ domains to estimate the internal consistency of the translated questionnaire. We considered a p-value of 0.05 and a 95% confidence interval (CI) to imply the statistical significance and precision of the results.

## Results

Sample characteristics

A total of 222 patients completed the survey, achieving a response rate of 31%. Twelve patients were excluded because they did not fulfill the inclusion criteria, resulting in 209 participants being enrolled in this study. The mean age was (Mean ± SD) 47.84 ± 10.8 years, with female patients comprising 63.1% of the sample. Tables 1, 2 present the sociodemographic and clinical characteristics and prevalence of comorbidities.

**Table 1:** Sociodemographic and clinical characteristics of the participants (N=209) ∓ = Standard deviation.

Item	Value
Age in years ∓SD	
Total	47.84 (∓10.2)
Male	47.75 (∓13.0)
Female	47.89 (∓8.5)
Gender, n (%)	
Male	69 (31.1%)
Female	140 (63.1%)
Marital status, n (%)	
Married	164 (78.5%)
Single	45 (21.5%)
Educational level, n (%)	
High school or lesser	37 (17.7%)
University or higher	172 (82.3%)
Occupational status, n (%)	
Unemployed	86 (41.1%)
Employed with no prolonged standing (<50% of a shift time)	47 (22.5%)
Employed with prolonged standing (>50% of a shift time)	76 (36.4%)
Monthly income (SAR), n (%)	
Low income (<6000)	58 (27.8%)
Middle income (6000-15000)	83 (39.7%)
High income (>15000)	51 (24.4%)
Exercise (Hours a week), n (%)	
Not exercising at all	97 (46.4%)
Exercising, (less than 2.5)	37 (17.7%)
Exercising, (more than 2.5)	75 (35.9%)
Footwear type, n (%)	
Walking shoe	113 (54.1%)
Boot - Ugg boot	1 (0.5%)
Slipper	49 (23.4%)
Backless slipper	10 (4.8%)
Sandal	10 (4.8%)
Oxford shoe	11 (5.3%)
High heel/Stiletto	1 (0.5%)
Court shoe - Mule	5 (2.4%)
Surgical/Bespoke footwear	0 (0%)
Moccassin	6 (2.9%)
Thong / Flipflop	3 (1.4%)
Type of footwear sole material, n (%)	
Rubber	118 (56.5%)
Plastic	44 (21.1%)
Leather	37 (17.7%)
Wood	4 (1.9%)

**Table 2:** Prevalence of comorbidities among male and female patients Notes: Percentages are representative of the total sample size (N=209)

Comorbodity, n (%)	Females (N=140)	Males (N=69)
Diabetes mellitus	27 (19.3%)	15 (25%)
Hypertension	29 (20.7%)	17 (28.3%)
Thyroid diseases	9 (6.4%)	1 (1.7%)
Dyslipidemia	5 (3.6%)	3 (5.0%)
Renal diseases	1 (0.07%)	0 (0.0%)
Heart diseases	4 (2.9%)	2 (3.3%)
Lung diseases	5 (3.6%)	0 (0.0%)
Osteoarthritis	1 (0.07%)	0 (0.0%)

Psychometric properties of the questionnaire

Cronbach's alpha was used to determine the internal consistency of the questionnaire (Table 3). The Arabic version of the foot-specific FHSQ section demonstrated high internal consistency, with values ranging from 0.809 to 0.954. Meanwhile, the generic FHSQ section was adopted from the validated Arabic version of the 36-item Short Form Survey (SF-36) with minor modifications, yielding acceptable Cronbach’s alpha values in this study (between 0.621 and 0.872).

**Table 3:** Cronbach’s alpha values for each domain of the Arabic version of the Foot Health Status Questionnaire CI = Confidence interval.

Domains (Questions covered)	Cronbach’s alpha coefficient	
	Value	CI 95%
Foot specific FHSQ		
Foot pain(1-4)	0.907	(0.885; 0.926)
Foot function(5-8)	0.906	(0.884; 0.926)
Footwear(10-12)	0.809	(0.759; 0.850)
General foot health(9+13)	0.954	(0.940; 0.965)
Generic FHSQ		
General health(14+19)	0.674	(0.560; 0.765)
Physical activity(15)	0.872	(0.844; 0.897)
Social activity(16+18)	0.76	(0.684; 0.818)
Vigour(17)	0.621	(0.470; 0.736)

Impact of sociodemographic and personal factors on participants' QoL

The FHSQ scores based on sociodemographic characteristics are presented in Table 4. For the foot-specific FHSQ section (Foot Pain, Foot Function, Footwear, and General Foot Health), unemployed participants and those with low income reported significantly lower scores (Median ± IQR), indicating poor QoL in the Foot Pain domain (unemployed: 41.2 ± 34.5; low-income: 35.6 ± 36.9; p < 0.05). Furthermore, individuals aged over 40 years and those with low income exhibited significantly greater QoL impairment in the General Foot Health domain (> 40 years: 60.0 ± 47.5; low-income: 42.5 ± 47.5; p < 0.05). No single factor revealed differences in the Foot Function or Footwear domains. In the generic FHSQ section, participants who did not exercise reported a significantly worse General Health domain score (60.0 ± 30.0, p < 0.05).

**Table 4:** Impact of sociodemographic and clinical characteristic on foot-specific and generic health-related quality of life using FHSQ scores. FHSQ = Foot Health Status Questionnaire, IQR = Interquartile range, SAR = Saudi Arabian Riyal Notes: Statistical significance was defined as p<0.05 (with a 95% confidence interval) in all analyses, with p-values obtained from the Kruskal-Wallis and Mann-Whitney U tests. * = statistically significant

Variables	n (%)	Foot specific FHSQ								Generic FHSQ							
		Foot pain		Foot function		Footwear		General foot health		General health		Physical activity		Social activity		Vigour	
		Median (IQR)	P-value	Median (IQR)	P-value	Median (IQR)	P-value	Median (IQR)	P-value	Median (IQR)	P-value	Median (IQR)	P-value	Median (IQR)	P-value	Median (IQR)	P-value
Age Group (years)																	
Less than 40	52 (24.9%)	53.4 (29.2)	0.32	75.0 (29.7)	0.27	41.7 (33.3)	0.33	60.0 (42.5)	0.04*	80.0 (30.0)	0.1	69.5 (38.9)	0.09	81.3 (37.5)	0.06	50.0 (18.8)	0.81
More than 40	145 (69.4%)	41.8 (30.6)		62.5 (43.8)		33.3 (41.7)		60.0 (47.5)		70.0 (30.0)		61.1 (38.9)		75.0 (50.0)		50.0 (18.8)	
Marital status																	
Married	164 (78.5%)	47.8 (35.5)	0.36	68.8 (43.8)	0.28	33.3 (33.3)	0.41	60.0 (47.5)	0.97	70.0 (40.0)	0.86	61.1 (37.5)	0.89	75.0 (37.5)	0.09	50.0 (25.0)	0.25
Single	45 (21.5%)	44.2 (30.3)		56.3 (37.5)		33.3 (33.3)		60.0 (60.0)		70.0 (30.0)		55.6 (50.0)		75.0 (43.8)		50.0 (25.0)	
Educational level																	
High school or lesser	37 (17.7%)	41.9 (36.6)	0.32	68.8 (46.9)	0.55	33.3 (41.7)	0.24	60.0 (53.8)	0.52	70.0 (30.0)	0.54	50.0 (36.1)	0.02*	75.0 (50.0)	0.35	50.0 (12.5)	0.48
University or higher	172 (82.3%)	48.1 (35.5)		62.5 (42.2)		33.3 (33.3)		60.0 (53.1)		70.0 (40.0)		61.1 (38.9)		75.0 (37.5)		50.0 (18.8)	
Occupational standing time																	
Unemployed	86 (41.1%)	41.2 (34.5)	0.04*	62.5 (37.5)	0.5	41.7 (41.7)	0.22	60.0 (38.1)	0.054	70.0 (40.0)	0.33	61.1 (38.9)	0.3	75.0 (50.0)	0.9	50.0 (18.8)	0.92
Employed with no prolonged standing (<50% of a shift time)	47 (22.5%)	48.1 (31.3)		62.5 (37.5)		25.0 (33.3)		60.0 (42.5)		70.0 (20.0)		66.7 (43.1)		75.0 (37.5)		50.0 (25.0)	
Employed with prolonged standing (>50% of a shift time)	76 (36.4%)	50.6 (35.5)		68.8 (43.8)		37.5 (33.3)		60.0 (60.0)		65.0 (37.5)		55.6 (44.4)		75.0 (37.5)		50.0 (18.8)	
Single Monthly income (SAR)																	
Low income (<6000)	58 (27.8%)	35.6 (36.9)	0.02*	59.4 (43.8)	0.17	41.7 (33.3)	0.48	42.5 (47.5)	0.02*	65.0 (40.0)	0.42	55.6 (38.9)	0.02*	75.0 (50.0)	0.39	50.0 (18.8)	0.1
Middle income (6000-15000)	83 (39.7%)	53.1 (26.9)		68.8 (31.3)		33.3 (33.3)		60.0 (42.5)		70.0 (30.0)		66.7 (44.4)		75.0 (37.5)		50.0 (18.8)	
High income (>15000)	51 (24.4%)	47.5 (43.1)		62.5 (50.0)		33.3 (41.7)		60.0 (47.5)		70.0 (30.0)		66.7 (44.4)		75.0 (37.5)		56.3 (25.0)	
Exercise (Hours per week)																	
Not exercising at all	97 (46.4%)	46.9 (30.9)	0.9	62.5 (37.5)	0.33	33.3 (33.3)	0.66	60.0 (47.5)	0.055	60.0 (30.0)	0.01*	55.6 (38.9)	0.01*	75.0 (43.8)	0.39	50.0 (25.0)	0.17
Exercising, (less than 2.5)	37 (17.7%)	47.5 (30.6)		68.8 (37.5)		33.3 (33.3)		60.0 (55.0)		80.0 (30.0)		66.7 (44.4)		75.0 (37.5)		50.0 (18.8)	
Exercising, (more than 2.5)	75 (35.9%)	48.1 (51.3)		62.5 (59.4)		41.7 (41.7)		60.0 (51.3)		70.0 (35.0)		61.1 (50.0)		75.0 (50.0)		50.0 (18.8)	

In the Physical Activity domain, differences were observed among those with high school or lower education levels (50.0 ± 36.1), low-income participants (55.6 ± 38.9), and those who did not exercise (55.6 ± 38.9) (p < 0.05). No single factor demonstrated differences in the Social Activity and Vigor domains. Neither footwear type nor sole material showed a statistically significant difference in any of the FHSQ domains.

Total scores and impact of gender on participants' QoL

Table 5 presents the results of the total FHSQ scores and the comparison between male and female participants. Regarding the foot-specific FHSQ section, the results demonstrated lower scores for women compared to men in all domains (p < 0.05) and lower QoL in terms of foot health for women. Additionally, in the generic FHSQ section, differences were found in the Physical Activity, Social Activity, and Vigor domains between men and women (p <0.05). Women had lower scores in these domains, indicating a lower QoL. No differences were observed in the General Health domain (p > 0.05).

**Table 5:** Impact of gender on health-related quality of life. FHSQ = Foot Health Status Questionnaire, SD = Standard deviation Notes: Statistical significance was defined as p<0.05 (with a 95% confidence interval) in all analyses. Measured using the FHSQ scores.

FHSQ domain	Total group mean ± SD (range), N = 209	Male mean ± SD (range), N = 69	Female mean ± SD (range), N = 140	P-value
Foot pain	47.36 ± 24.38	55.52 ± 53.75	43.34 ± 24.10	0.001
	(0–85)	(0–85)	(0–81)	
Foot function	61.48 ± 26.27	71.92 ± 25.47	56.34 ± 25.20	0
	(0–100)	(18–93)	(0–100)	
Footwear	37.84 ± 24.20	47.83.01 ± 24.99	32.92 ± 22.30	0
	(0–100)	(0–100)	(0–100)	
General foot health	53.98 ± 29.59	62.46 ± 27.42	48.48 ± 29.62	0.001
	(0–100)	(0–100)	(0–100)	
General health	68.33 ± 22.22	70.43 ± 21.72	67.29 ± 22.47	0.338
	(0–100)	(0–100)	(0–100)	
Physical activity	60.13 ± 25.63	69.81 ± 25.54	55.36 ± 24.38	0
	(0–100)	(33–100)	(0–88)	
Social capacity	74.40 ± 23.80	82.79 ± 20.24	70.27 ± 24.38	0
	(0–100)	(0–100)	(0–100)	
Vigor	48.80 ± 15.89	54.17 ± 16.03	46.16 ± 15.19	0
	(0–100)	(12–100)	(0–100)	

## Discussion

This study assessed the extent of foot-specific and generic HRQoL impairment among individuals diagnosed with PF in Saudi Arabia and the difference in its impact between male and female patients using FHSQ scores; only one previous study had demonstrated this impact. However, such studies are yet to be conducted in the Middle East or Asia region [7]. The FHSQ scores and other sociodemographic and footwear factors were obtained to determine their influence on the HRQoL of patients with PF. The prevalence of PF among women in this study was 2.0 times higher than that among men, which partially echoes the outcome of a large investigation carried out in the United States, which estimated that female participants are 2.5 times more likely than male participants to suffer from this condition [9]. A previous study conducted in Makkah, Saudi Arabia, found that a staggering 43.3% of patients diagnosed with PF were female among a sample of patients who visited primary care clinics complaining of heel pain [24]. Among the various factors analyzed, lower-income participants exhibited the most diminished scores (indicating a poorer QoL) in most of the FHSQ domains. Significant differences in Foot Pain, General Foot Health, and Physical Activity were observed. However, the literature has yet to highlight this intriguing association. This study addresses the gap in existing research by highlighting the role of personal income as a major contributing factor to QoL in individuals suffering from PF. Some studies have suggested the role of income as a risk factor for the development of foot-related issues; however, this connection remains a topic of contention, as other studies have failed to establish a similar link [25-28].

These findings should be considered with the fact that the main treatment of PF is conservative management which includes physiotherapy and footwear replacement. This can explain how income can impact foot health because the recommended footwear is limited in number, and most of them are highly branded and high-priced which are not covered by insurance or government plans. This necessitates the discovery of affordable and available alternatives to overcome this issue such as Extracorporeal shock wave therapy was shown to have a good effect on improving foot pain and function in PF patients [29]. To achieve success in physiotherapy, it is important to emphasize adherence to a daily routine and highlight the significance of stretching the gastrocnemius muscle, which is a fundamental aspect of treating PF [30]. This association could be stronger for unemployed patients considering the Foot Pain domain because financial constraints resulting from unemployment may limit individuals' ability to access appropriate footwear or seek timely medical care for foot-related problems [31].

Furthermore, our study findings revealed that participants aged >40 years have more impaired QoL in the General Foot Health domain, even though they have the same median QoL as those aged <40 years old; this indicates a significant difference in the distribution, which is implicated in the IQR [32]. Foot health issues can affect people of all ages, but older individuals may experience more problems due to decreased mobility, aging, and conditions like arthritis and diabetes. In addition, the study found a direct relationship between diabetes and worse outcomes in all foot health domains except Footwear and Vigor. Other studies have also found that diabetes contributes to poorer foot health, which may explain the poorer General Foot Health score among older people in our study [33,34]. Similarly, this study found that higher education level and regular exercise were associated with a better QoL related to the Physical Activity domain. People with more education tend to have better access to health information, appreciate the benefits of exercise more, and possess the knowledge and skills to remain physically active. Moreover, higher education can provide individuals with the knowledge and skills to engage in activities that promote physical well-being [35,36]. The results of this study revealed that PF significantly impacts both foot-specific and generic HRQoL, with lower scores observed in all domains except Social Capacity when compared to a recent study on the general population in Saudi Arabia using the FHSQ [37]. These results are in line with other studies employing the FHSQ among PF patients [7,11,12,38]. Although, in our study, the Footwear domain had a particularly heavy impact in comparison, which may reflect cultural shoe-wearing practices and/or difficulty finding appropriate and affordable footwear. Conversely, the General Foot Health domain was less affected in our sample, which may be attributed to an adaptive response to this foot condition. This is particularly evident as we included patients with active disease status diagnosed in 2016, in contrast to previous studies that included patients attending the clinic. Adaptation to chronic conditions is a well-documented phenomenon that can introduce bias in subjective measures [39]. Ultimately, our findings provide insight into how PF impacts QoL, and comparisons with other studies should be made cautiously due to criteria and methodological variations. However, the FHSQ does not have a single total score. Therefore, a comparison with other diseases could be difficult because it is necessary to interpret every score in the eight domains. However, compared to other studies that adopted the FHSQ, PF generally affects foot-specific QoL more than hyperkeratosis, foot arch height, Parkinson's disease, Alzheimer’s disease, breast cancer, asthma, hemophilia, and diabetes mellitus, and is comparable to hallux valgus degree 3, with foot pain and foot function being more impacted than lesser toe deformity. Diseases such as fibromyalgia had a greater impact in both sections of the FHSQ than PF, as observed in our sample [34,40-49]. The outcomes of our study indicate that female participants exhibited lower scores on the FHSQ than male participants, which is in line with a previous study [7]. This difference between men and women has also been demonstrated in the general population in Saudi Arabia [37]. Consequently, female patients with PF are more prone to experiencing poorer specific and general QoL concerning foot health; this disparity in QoL was attributed to variations in factors such as footwear choices, pain severity, engagement in physical activities, or social characteristics between male and female patients [7,37,50]. This research has various strengths, including a considerable population, examination of previously unassessed factors such as footwear choice, sports participation, and sociodemographic characteristics, and inclusion of a broad spectrum of old and newly diagnosed patients, regardless of clinical follow-up. This provides an opportunity to examine adaptation as a confounding factor that should be further evaluated in future research. However, this study has several limitations. First, we used an online questionnaire through WhatsApp, which prevented the collection of clinical characteristics such as weight, height, BMI, and structural foot differences. Obtaining the BMI by file number from the E-SIHI system was not possible because the data may be outdated. Second, using the Arabic version of the FHSQ is a potential limitation; although it has high internal consistency, it is still not a validated tool. Third, our findings stem from a cross-sectional and exploratory design, which means that the results should be considered hypothesis-generating rather than conclusive evidence of causal or temporal connections between QoL domains and associated characteristics. Finally, while statistical analysis was used, a comprehensive interpretation of the data may require a combination of visual and statistical analyses. Further studies are strongly recommended to contribute to a better appreciation of the variables that can enhance QoL across diverse cultures and demographics. Additionally, incorporating a pain scale such as the Visual Analogue Scale (VAS 100 mm score) and correlating it with QoL scores and the date of diagnosis could yield valuable insights and add significant value to the existing literature. Further research is necessary to understand the underlying mechanisms in these particular areas and to study the impact of sex on therapy outcomes.

## Conclusions

QoL was impaired for patients with low income, aged above 40 years, having lower educational levels, not engaging in exercise, and unemployed with regard to Foot Pain, General Foot Health, General Health, and Physical Activity domains. Moreover, female patients showed a more impaired QoL in all FHSQ domains except General Health, with Footwear being the most affected domain compared to global studies. Therefore, we recommend paying particular attention to the footwear domain when assessing and treating patients with PF. The lack of appropriate and affordable footwear options may be a significant barrier to the effective management of PF in Saudi Arabia, especially among female patients. We also recommend recognizing the role of chronic diseases, such as diabetes mellitus, as a contributing factor that may impair foot health and QoL.

## References

[1] Ficke J, Byerly DW (2023). Anatomy, Bony Pelvis and Lower Limb: Foot.

[2] Buchanan BK, Kushner D (2022). Plantar Fasciitis.

[3] Phillips A, McClinton S (2017). Gait deviations associated with plantar heel pain: a systematic review. Clin Biomech (Bristol, Avon).

[4] Ríos-León M, Valera-Calero JA, Ortega-Santiago R, Varol U, Fernández-de-Las-Peñas C, Plaza-Manzano G (2022). Analyzing the interaction between clinical, neurophysiological and psychological outcomes underlying chronic plantar heel pain: a network analysis study. Int J Environ Res Public Health.

[5] Rhim HC, Kwon J, Park J, Borg-Stein J, Tenforde AS (2021). A systematic review of systematic reviews on the epidemiology, evaluation, and treatment of plantar fasciitis. Life (Basel).

[6] DeMaio M, Paine R, Mangine RE, Drez D Jr (1993). Plantar fasciitis. Orthopedics.

[7] Palomo-López P, Becerro-de-Bengoa-Vallejo R, Losa-Iglesias ME, Rodríguez-Sanz D, Calvo-Lobo C, López-López D (2018). Impact of plantar fasciitis on the quality of life of male and female patients according to the Foot Health Status Questionnaire. J Pain Res.

[8] van Leeuwen KD, Rogers J, Winzenberg T, van Middelkoop M (2016). Higher body mass index is associated with plantar fasciopathy/'plantar fasciitis': systematic review and meta-analysis of various clinical and imaging risk factors. Br J Sports Med.

[9] Barton CJ, Bonanno D, Menz HB (2009). Development and evaluation of a tool for the assessment of footwear characteristics. J Foot Ankle Res.

[10] Gutteck N, Schilde S, Delank KS (2019). Pain on the plantar surface of the foot. Dtsch Arztebl Int.

[11] Irving DB, Cook JL, Young MA, Menz HB (2008). Impact of chronic plantar heel pain on health-related quality of life. J Am Podiatr Med Assoc.

[12] Landorf KB, Kaminski MR, Munteanu SE, Zammit GV, Menz HB (2023). Activity and footwear characteristics in people with and without plantar heel pain: a matched cross-sectional observational study. Musculoskeletal Care.

[13] Bennett PJ, Patterson C, Wearing S, Baglioni T (1998). Development and validation of a questionnaire designed to measure foot-health status. J Am Podiatr Med Assoc.

[14] Martin RL, Irrgang JJ (2007). A survey of self-reported outcome instruments for the foot and ankle. J Orthop Sports Phys Ther.

[15] Landorf KB, Keenan AM (2002). An evaluation of two foot-specific, health-related quality-of-life measuring instruments. Foot Ankle Int.

[16] Kish Kish, L L (1995). Survey sampling. https://psycnet.apa.org/record/1965-35018-000.

[17] Menon V, Muraleedharan A (2020). Internet-based surveys: relevance, methodological considerations and troubleshooting strategies. Gen Psychiatr.

[18] Beaton DE, Bombardier C, Guillemin F, Ferraz MB (2000). Guidelines for the process of cross-cultural adaptation of self-report measures. Spine (Phila Pa 1976).

[19] Irving DB, Cook JL, Menz HB (2006). Factors associated with chronic plantar heel pain: a systematic review. J Sci Med Sport.

[20] Werner RA, Gell N, Hartigan A, Wiggerman N, Keyserling WM (2010). Risk factors for plantar fasciitis among assembly plant workers. PM R.

[21] Waclawski ER, Beach J, Milne A, Yacyshyn E, Dryden DM (2015). Systematic review: plantar fasciitis and prolonged weight bearing. Occup Med (Lond).

[22] Anderson J, Granat MH, Williams AE, Nester C (2019). Exploring occupational standing activities using accelerometer-based activity monitoring. Ergonomics.

[23] Matthias EC, Banwell HA, Arnold JB (2021). Methods for assessing footwear comfort: a systematic review. Footwear Sci.

[24] Goweda R, Alfalogy E, Filfilan R, Hariri G (2015). Prevalence and risk factors of plantar fasciitis among patients with heel pain attending primary health care centers of Makkah, Kingdom of Saudi Arabia. J High Institute Public Health.

[25] Menz HB (2016). Chronic foot pain in older people. Maturitas.

[26] Greenberg L, Davis H (1993). Foot problems in the US. The 1990 National Health Interview Survey. J Am Podiatr Med Assoc.

[27] Barr EL, Browning C, Lord SR, Menz HB, Kendig H (2005). Foot and leg problems are important determinants of functional status in community dwelling older people. Disabil Rehabil.

[28] Chen J, Devine A, Dick IM, Dhaliwal SS, Prince RL (2003). Prevalence of lower extremity pain and its association with functionality and quality of life in elderly women in Australia. J Rheumatol.

[29] Melese H, Alamer A, Getie K, Nigussie F, Ayhualem S (2022). Extracorporeal shock wave therapy on pain and foot functions in subjects with chronic plantar fasciitis: systematic review of randomized controlled trials. Disabil Rehabil.

[30] Latt LD, Jaffe DE, Tang Y, Taljanovic MS (2020). Evaluation and treatment of chronic plantar fasciitis. Foot Ankle Orthop.

[31] Huang J, Birkenmaier J, Kim Y (2014). Job loss and unmet health care needs in the economic recession: different associations by family income. Am J Public Health.

[32] Hart A (2001). Mann-Whitney test is not just a test of medians: differences in spread can be important. BMJ.

[33] Domínguez-Muñoz FJ, Garcia-Gordillo MA, Diaz-Torres RA (2020). Foot health status questionnaire (FHSQ) in Spanish people with type 2 diabetes mellitus: preliminary values study. Int J Environ Res Public Health.

[34] López-López L, Losa-Iglesias ME, Gómez-Salgado J (2022). The implications of diabetic foot health-related with quality of life: a retrospective case control investigation. J Tissue Viability.

[35] Vandelanotte C, Duncan MJ, Stanton R (2019). Validity and responsiveness to change of the Active Australia Survey according to gender, age, BMI, education, and physical activity level and awareness. BMC Public Health.

[36] Buchmann M, Jordan S, Loer AM, Finger JD, Domanska OM (2023). Motivational readiness for physical activity and health literacy: results of a cross-sectional survey of the adult population in Germany. BMC Public Health.

[37] Almaawi A, Alqarni H, Thallaj AK, Alhuqbani M, Aldosari Z, Aldosari O, Alsaber N (2023). Foot health and quality of life among adults in Riyadh, Saudi Arabia: a cross-sectional study. J Orthop Surg Res.

[38] Okur SÇ, Aydın A (2019). Comparison of extracorporeal shock wave therapy with custom foot orthotics in plantar fasciitis treatment: a prospective randomized one-year follow-up study. J Musculoskelet Neuronal Interact.

[39] Groot W (2000). Adaptation and scale of reference bias in self-assessments of quality of life. J Health Econ.

[40] López-López D, Painceira-Villar R, Becerro-de-Bengoa-Vallejo R, Losa-Iglesias ME, Rodríguez-Sanz D, Palomo-López P, Calvo-Lobo C (2018). Impact of the mechanical hyperkeratotic lesions and its association with quality of life: an observational case-control study. J Eur Acad Dermatol Venereol.

[41] López López D, Bouza Prego Mde L, Requeijo Constenla A, Saleta Canosa JL, Bautista Casasnovas A, Tajes FA (2014). The impact of foot arch height on quality of life in 6-12 year olds. Colomb Med (Cali).

[42] López-López D, Grela-Fariña M, Losa-Iglesias ME, Calvo-Lobo C, Rodríguez-Sanz D, Palomo-López P, Becerro-de-Bengoa-Vallejo R (2018). Clinical aspects of foot health in individuals with Alzheimer’s disease. Int J Environ Res Public Health.

[43] Navarro-Flores E, Jiménez-Cebrián AM, Becerro-de-Bengoa-Vallejo R (2022). Effect of foot health and quality of life in patients with Parkinson disease: a prospective case-control investigation. J Tissue Viability.

[44] Palomo-López P, Rodríguez-Sanz D, Becerro-de-Bengoa-Vallejo R, Losa-Iglesias ME, Guerrero-Martín J, Calvo-Lobo C, López-López D (2017). Clinical aspects of foot health and their influence on quality of life among breast cancer survivors: a case-control study. Cancer Manag Res.

[45] López DL, González LC, Iglesias MEL, Canosa JL, Sanz DR, Lobo CC, de Bengoa Vallejo RB (2016). Quality of life impact related to foot health in a sample of older people with hallux valgus. Aging Dis.

[46] López-López D, Martínez-Vázquez M, Losa-Iglesias ME, Calvo-Lobo C, Rodríguez-Sanz D, Palomo-López P, Becerro-de-Bengoa-Vallejo R (2018). Foot health-related quality of life among elderly with and without lesser toe deformities: a case-control study. Patient Prefer Adherence.

[47] Palomo-López P, Calvo-Lobo C, Becerro-de-Bengoa-Vallejo R, Losa-Iglesias ME, Rodriguez-Sanz D, Sánchez-Gómez R, López-López D (2019). Quality of life related to foot health status in women with fibromyalgia: a case-control study. Arch Med Sci.

[48] Jiménez-Cebrián AM, López-López D, Becerro-de-Bengoa-Vallejo R (2020). Foot health-related quality of life in hemophiliacs: a case-control study. Int J Med Sci.

[49] López-López D, Painceira-Villar R, García-Paz V, Becerro-de-Bengoa-Vallejo R, Losa-Iglesias ME, Rodríguez-Sanz D, Calvo-Lobo C (2019). Impact of the allergic asthma on foot health-related quality of life and depression: a novel case-control research. Medicina (Kaunas).

[50] Katsambas A, Abeck D, Haneke E, van de Kerkhof P, Burzykowski T, Molenberghs G, Marynissen G (2005). The effects of foot disease on quality of life: results of the Achilles Project. J Eur Acad Dermatol Venereol.

